# Tunable, permanent and instantly available super-wettability states on metal surfaces by laser texturing and plasma coating

**DOI:** 10.1038/s41598-025-11750-z

**Published:** 2025-07-29

**Authors:** Daniel Holder, Paul Reichle, Georg Umlauf, Rudolf Weber, Jakob Barz, Thomas Graf

**Affiliations:** 1 Institut für Strahlwerkzeuge (IFSW), University of Stuttgart, Stuttgart, Germany; 2 ARENA2036 Research Campus, Stuttgart, Germany; 3 Institute of Interfacial Process Engineering and Plasma Technology (IGVP), University of Stuttgart, Stuttgart, Germany; 4 Fraunhofer Institute for Interfacial Engineering and Biotechnology, Stuttgart, Germany; 5 Fraunhofer Institute for Electronic Nano Systems, Chemnitz, Germany

**Keywords:** Wettability, Metals, Surface structures, Laser texturing, Plasma coating, Plasma-enhanced chemical vapor deposition, Superhydrophilic, Superhydrophobic, Mechanical engineering, Ultrafast lasers, Surface chemistry

## Abstract

Wettability, the ability of a liquid to spread on or repel from a surface, holds particular significance for applications requiring extreme control of liquid interaction, including self-cleaning, anti-icing, dropwise condensation, anti-fogging, and enhanced fluid transport. This work investigates the synergistic combination of laser surface texturing and plasma-enhanced chemical vapor deposition (PECVD) to achieve tunable, permanent, and instantly available super-wettability states on metal surfaces. Ultrashort laser pulses were employed to produce various surface textures, ranging from fine nanoscale ripples to rougher microtextures such as microgrooves, spikes, and holes, on stainless steel AISI 304, copper, and the titanium alloy Ti64. PECVD coatings, including thin layers of glass and polymers, were subsequently applied to these textures to modulate surface chemistry and achieve the desired wettability.The results demonstrate that superhydrophilic surfaces with a water contact angle *θ* < 10° were achieved by combining rough textures with thin glass coatings, offering long-term stability that could be simply renewed via ultrasonic cleaning. Conversely, superhydrophobic surfaces with a water contact angle *θ* > 150° were instantly obtained using polymer coatings on rough textures. These functionalized surfaces also exhibited exceptional liquid repellence for complex liquids, such as milk and beer, making them particularly suitable for special applications using solutions or emulsions. The integration of laser texturing and PECVD coating provides a versatile and simple pathway for fabricating functional surfaces with tunable wettability and long-term performance across multiple metals and fluids.

## Introduction

Surface functionalization has emerged as a strategy for tailoring surface properties to meet the demands of advanced applications in diverse fields such as aerospace, biomedical devices, energy systems, or consumer electronics. By modifying surface characteristics, properties such as wettability, optical reflectivity, and tribological behaviour can be tuned to enhance the performance of a surface and enable novel functionalities^1^. Among these properties, wettability - the ability of a liquid to spread on or repel from a surface - holds particular significance for applications requiring extreme control of liquid interaction, including self-cleaning, anti-icing, biofilm inhibition, and enhanced fluid transport^2,3^.

The wettability of a surface is determined by the interaction of its chemical composition and micro-/nanoscale topography^4^. Wetting behavior is often described using classical models such as the Wenzel^5^ and Cassie-Baxter^6^ model. The Wenzel model assumes that a liquid completely penetrates the surface texture, leading to increased contact area between the liquid and the substrate, thereby amplifying the inherent wetting properties of the material^5^. In contrast, the Cassie-Baxter model describes a state where liquid bridges across surface roughness, trapping gas beneath and resulting in a composite interface^6^. These models provide theoretical frameworks for understanding and engineering surfaces to achieve either superhydrophilic or superhydrophobic states.

Surfaces with superhydrophilic properties, characterized by extremely low water contact angles (typically *θ* < 10°), find applications in areas such as anti-fogging surfaces^7,8^, lubrication^9^, fluid transport^10–13^ and fluid absorption in filtration systems^14–16^. Conversely, superhydrophobic surfaces, with water contact angles *θ* > 150°, are critical in applications requiring low wettability, such as self-cleaning^17–20^, corrosion-resistant^21–23^, and drag-reducing^24,25^ surfaces in marine environments, and surfaces exhibiting dropwise condensation for enhanced heat transfer in cooling systems^26–28^.

Metals such as stainless steel, copper, and titanium alloys are extensively used across industries due to their mechanical robustness, corrosion resistance, or thermal conductivity. However, these metals or their surface oxide layer are intrinsically hydrophilic, often showing water contact angles in the range of 50° to 80°^4,29,30^. To enable application-specific wettability, surface functionalization techniques become essential.

Surface texturing with ultrafast lasers has emerged as a precise and versatile tool for engineering surface topography at micro- and nanoscales. Ultrafast lasers operate with extremely short pulse durations in the range of femtoseconds to picoseconds. This minimizes heat-affected zones and preserves the material’s bulk properties during surface texturing. By tailoring processing parameters such as fluence^31–33^, repetition rate^33^ and scanning speed^26,29,34^, laser-induced periodic surface structures (LIPSS)^32,35–37^ or hierarchical structures with micro-/nano-roughness^29,31,38,39^ can be textured onto large areas^40,41^, which plays a critical role in manipulating the wettability of metal surfaces. However, the stability of the induced wetting states can pose challenges. While laser-induced textures can provide long-term stability in terms of topographical features, their wettability may degrade over time due to surface contamination or oxidation, especially in superhydrophilic states^4,38,42,43^.

Plasma-enhanced chemical vapor deposition (PECVD) is a coating technique widely employed to modify surface chemistry by depositing thin films under low-pressure plasma conditions^44^. In this process, a precursor gas is ionized into plasma, and the active species interact with the surface of the substrate to form a chemically bonded layer^45^. The PECVD coating is also characterized by high gap mobility and layer homogeneities with simultaneous low possible coating thicknesses (*d* < 1 µm), ensuring minimal alteration to the surface topography. PECVD is particularly advantageous for adjusting the surface’s chemical functionality, which directly influences wettability. For instance, hydrophilic or hydrophobic coatings can be achieved by introducing oxygen-containing or fluorinated precursors, respectively^46–48^.

The combination of surface textures and coatings has already been used by various research groups to enhance a wide range of applications. For instance, this approach has been employed to improve the replication ratio of injection-molded plastic parts^49^, reduce wear and friction in plastic components^50^, and promote high rates of cellular proliferation^51^. Significant progress has also been made in the field of surface wetting. Nanosecond laser texturing combined with in-situ SiO_2_ deposition or laser-micromachined grooves coated with a Na-based zeolite were used to attain stable superhydrophilic surfaces^43,52^. Nanosecond laser texturing was also combined with a FAS-TiO_2_/epoxy coating in order to obtain instant superhydrophobic surfaces with enhanced corrosion resistance against aggressive media^53^. Superhydrophobic surfaces have also been developed with coatings that are more environmentally friendly than fluorinated (PFAS) coatings^54^. When combined with the appropriate chemical composition, directional surface patterns such as grooves have demonstrated the potential to achieve anisotropic wetting behaviour^55^.

While these advances highlight the potential of integrating surface texturing with coatings, most existing approaches face limitations in texturing rate, long-term stability, or the ability to achieve dynamically tunable wettability. This work addresses these challenges by developing and characterizing metal surfaces that exhibit instantly available, adjustable, extreme (“super-”) and long-term stable wettability under ambient storage. By combining ultrafast laser texturing with plasma-enhanced chemical vapor deposition, we introduce a synergistic approach that surpasses the individual constraints of each technique. This method not only enables precise control over surface wetting properties but also provides a scalable and straightforward route to engineering functional metal surfaces for demanding applications requiring extreme wetting characteristics.

## Materials and methods

**Fig. 1:**
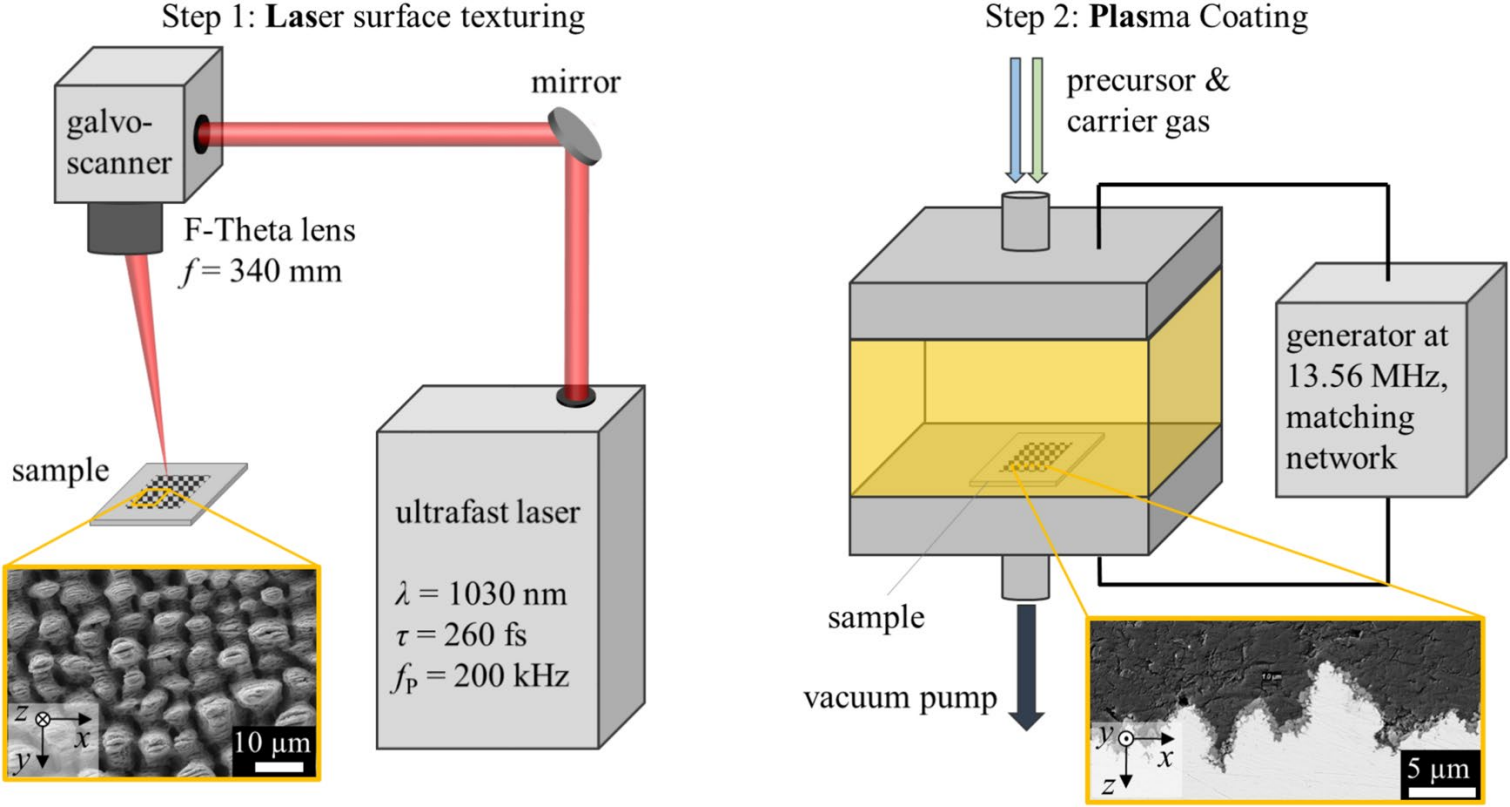
Experimental setup for the LasPlas process, i.e. laser surface texturing of metallic surfaces with an ultrafast laser (left) and subsequent plasma coating using PECVD (right).

Figure 1 shows the sequential combination of laser surface texturing (“Las”) and plasma coating (“Plas”) used in this work, hereafter also referred to as “LasPlas” process.

In step 1, the surface of the sample is textured by irradiation with ultrashort laser pulses. Figure 1 (left) shows the experimental setup used for laser surface texturing with an ultrafast laser (*Pharos, Light Conversion*). The ultrafast laser delivered pulses with a pulse duration of *τ* = 260 fs at a repetition rate of *f*_P_ = 200 kHz. The linearly polarized beam with a wavelength of *λ* = 1030 nm was guided via mirrors to a galvanometer scanner (*intelliSCAN 30, Scanlab*). Using an F-Theta lens with a focal length of *f* = 340 mm (*Sill Optics*), the beam was focused onto the workpiece surface with a diameter of *d*_0_ = 120 ± 5 µm. Squared areas with an edge length of 10 mm were textured in ambient atmosphere by scanning the surface with the focused laser beam. The peak fluence (adjusting the pulse energy) and the scanning speed were varied to produce different surface textures. The productivity of texturing was expressed using the texturing rate

1 $$\dot{A} = \frac{{v_{x} \cdot p_{y} }}{n},$$

where *v*_*x*_ and *p*_*y*_ are the scanning speed and line hatching distance, respectively, and *n* is the number of scans over the surface. The surface was scanned using unidirectional scanning paths with a constant line hatching distance of *p*_*y*_ = 24 µm, which corresponds to a line overlap of 80%. Previous studies have shown that a homogeneous surface morphology can be produced with this value of line overlap^26^.

In step 2, PECVD is used to generate a coating on the previously textured surfaces (cf. Fig. 1 right), which are not cleaned between laser texturing and plasma coating. The surfaces were coated with either a silicone-like plasma polymer coating (PP), a polytetrafluorethylen (PTFE) coating or a glass (SiO_x_) coating approximately one hour after laser texturing. The basic pressure and the process pressure in the deposition chamber were set to 0.005 mbar and 0.1 mbar, respectively. As precursors, hexamethyldisiloxane-C_6_H_18_OSi_2_ (HMDSO) was used for the silicone-like polymer coating and glass coating. Octafluorocyclobutane-C_4_F_8_ was used for the PTFE coating. Argon was used as carrier and activation gas. Depending on the coating, nitrogen (for PP) or oxygen (for SiO_x_) were used as process gas with a flow rate in the in the range of 50 to 150 sccm (standard cm^3^/min). Depending on the type of coating, the plasma power varied between 80 and 300 W using a high-frequency generator (*Advanced Energy, Cesar*) at 13.56 MHz with corresponding matching network (*Advanced Energy, Navio*). The samples heat up from room temperature by approximately 2 K/min process time during coating with a deposition rate of approximately 60 nm/min. The thickness was measured by a reference measurement using ellipsometry (*Sentech, SE801*) on polished silicon wafers and regularly checked by scanning electron microscopy (SEM) or atomic force microscopy (AFM). Either thin coatings with a thickness in the order of 90 to 160 nm or thick coatings with a thickness in the order of 1000 to 1110 nm were produced.

The majority of the tests were carried out with AISI 304 stainless steel, which is used in the food, automotive and chemical industries due to its high formability and corrosion resistance^56^. The polished samples had an initial roughness of *S*_a_ = 0.03 µm before further processing. Further tests were carried out with copper and the titanium alloy Ti64. Copper is used in electro mobility, piping sectors and marine due to its high electrical and thermal conductivity and anti-fouling properties^57^. Ti64 is often used in medical technology and aerospace due to its biocompatibility, high corrosion resistance and high strength-to-weight ratio^58^. The rolled samples each had an initial roughness of *S*_a_ = 0.39 µm and *S*_a_ = 0.64 µm respectively prior to further processing. All samples had a thickness between 1 and 3 mm.

All measurements except for SEM were performed in ambient atmosphere at room temperature. The topographical characterization of the textured samples was carried out using a laser scanning microscope LSM (*Keyence, VK-9710-K*) and SEM (*Zeiss GeminiSEM 500* and *Jeol JSM-6490LV*). The roughness

2 $$S_{a} = \frac{1}{M \cdot N}\sum\limits_{m = 1}^{M} {\sum\limits_{n = 1}^{N} {\left| {z\left( {x_{m} ,y_{n} } \right) - \left\langle z \right\rangle } \right|} } $$

was used to quantify the surface topography in one value, where *z* is the height measured at the coordinates *x* and *y,* and where *M* and *N* are the number of measured points along the coordinates, respectively. The (static) contact angle was measured in using the camera-based sessile drop method (*DataPhysics, OCA 15 EC*) with droplets of deionized water (DI water) with a volume between 1 and 10 µℓ. The image for measuring the contact angle was taken approximately 3 s after droplet application. The camera-based measurements of the roll-off and spreading behavior were performed using DI water with a droplet volume of 10 µℓ. The samples were stored in a glass container with a loose-fitting lid, thus ensuring that the storage was not airtight. However, there was no open exposure to dust and particles in the ambient air.

## Topographical characterization of steel surfaces treated by laser texturing and plasma coating

**Fig. 2:**
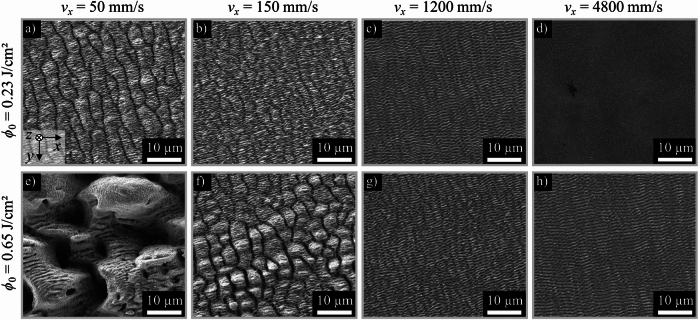
SEM images of surface textures after laser texturing with different peak fluences and scanning speeds on stainless steel AISI 304. *λ* = 1030 nm, *τ* = 260 fs, *f*_P_ = 200 kHz, *d*_0_ = 120 ± 5 µm, *p*_*y*_ = 24 µm,* n* = 1.

Figure 2 shows SEM images of stainless steel surfaces textured with different peak fluences *ϕ*_0_ of 0.23 J/cm^2^ and 0.65 J/cm^2^ and varying scanning speeds *v*_*x*_ in the range of 50 mm to 4800 mm/s.

At the same scanning speeds, lower peak fluences (achieved with lower pulse energies and hence lower average power) lead to finer structures than higher peak fluences (obtained with higher pulse energies and hence higher average power). Low scanning speeds in the range of 50 mm/s ≤ *v*_*x*_ ≤ 150 mm/s produce rough structures as the surface is irradiated with a high number of overlapping pulses. This roughening can be seen in the form of microgrooves formed perpendicular to the orientation of the LIPSS (Fig. 2a, f). When the irradiation exceeds levels that prevent the surface temperature from falling below melting point due to heat accumulation between pulses, spikes (or spikes & holes) are formed^34^, resulting in much rougher structures with a higher spatial period in the range of tens of microns (Fig. 2e). This rough surface is often covered with the finer LIPSS, which means that hierarchical structures can be generated simply by scanning the surface with low scanning speeds at moderate peak fluences that significantly exceed the ablation threshold. At scanning speeds of *v*_*x*_ = 1200 mm/s and higher, ripple-shaped structures are produced on the surface. These LIPSS are low spatial frequency LIPSS (LSFL) with a spatial period of around 0.9 µm. With the peak fluence of *ϕ*_0_ = 0.23 J/cm^2^ and the highest investigated scanning speed *v*_*x*_ = 4800 mm/s, fine LIPSS with a spatial period of only 0.2 µm were fabricated on the surface (Fig. 2d), which are also referred to as high spatial frequency LIPSS (HSFL)^36^.

**Fig. 3:**
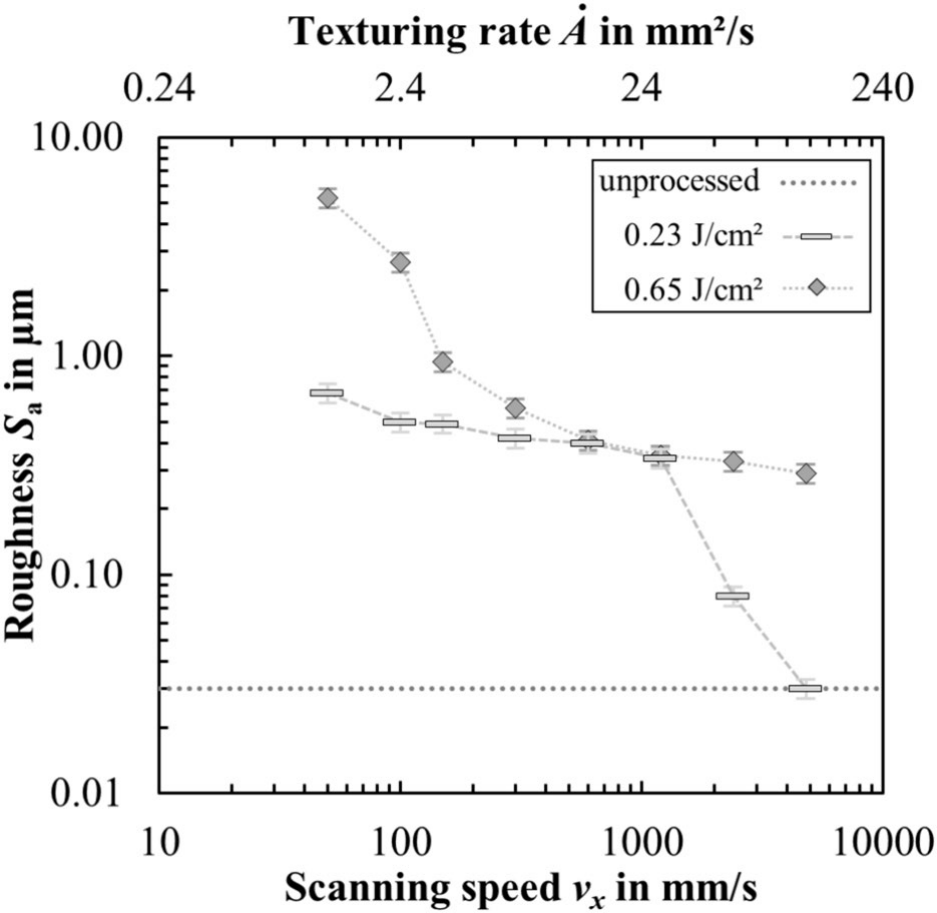
Measured surface roughness *S*_a_ of the laser textured steel samples as a function of scanning speed *v*_*x*_ and texturing rate $\dot{A}$. The roughness of the unprocessed initial surface is shown as a dotted line.

Figure 3 shows the corresponding roughness values *S*_a_ of textured surfaces as a function of the scanning speed *v*_*x*_. The roughness value of the unprocessed initial surface is shown as a dotted line.

Figure 3 shows that low scanning speeds lead to the surfaces with higher roughness values, which is in good agreement with the surface morphologies shown in Fig. 2. As the scanning speed increases, the roughness continues to decrease and approaches the initial roughness value of the unprocessed sheets. At the same scanning speeds, a lower peak fluence (Fig. 3, rectangle) generally leads to lower roughness values than higher peak fluences (Fig. 3, diamonds). These results agree well with results presented in other works^4,26,38,59^. Depending on the roughness range, different textures could be observed. For roughness values *S*_a_ > 1.5 µm, spikes dominate the surface morphology, some of which are still covered with ripples. In the range of 0.5 µm < *S*_a_ < 1.5 µm, the surface is determined by LSFL and microgrooves. Only LSFL were observed in the range of 0.3 µm < *S*_a_ < 0.5 µm. The roughness of the surface textured with HSFL did not deviate from the surface roughness of the polished sample, which was measured to be *S*_a_ = 0.03 µm. Different textures and degrees of roughness can be obtained by the choice of scanning speed *v*_*x*_ and peak fluence *ϕ*_0_. The texturing rate $\dot{A}$, i.e. the achievable productivity when creating different structures, is directly linked to the scanning speed *v*_*x*_ due to the relationship shown in Eq. (1). Therefore, the highest texturing rates can be achieved with the fine ripples (LSFL and HSFL).

**Fig. 4:**
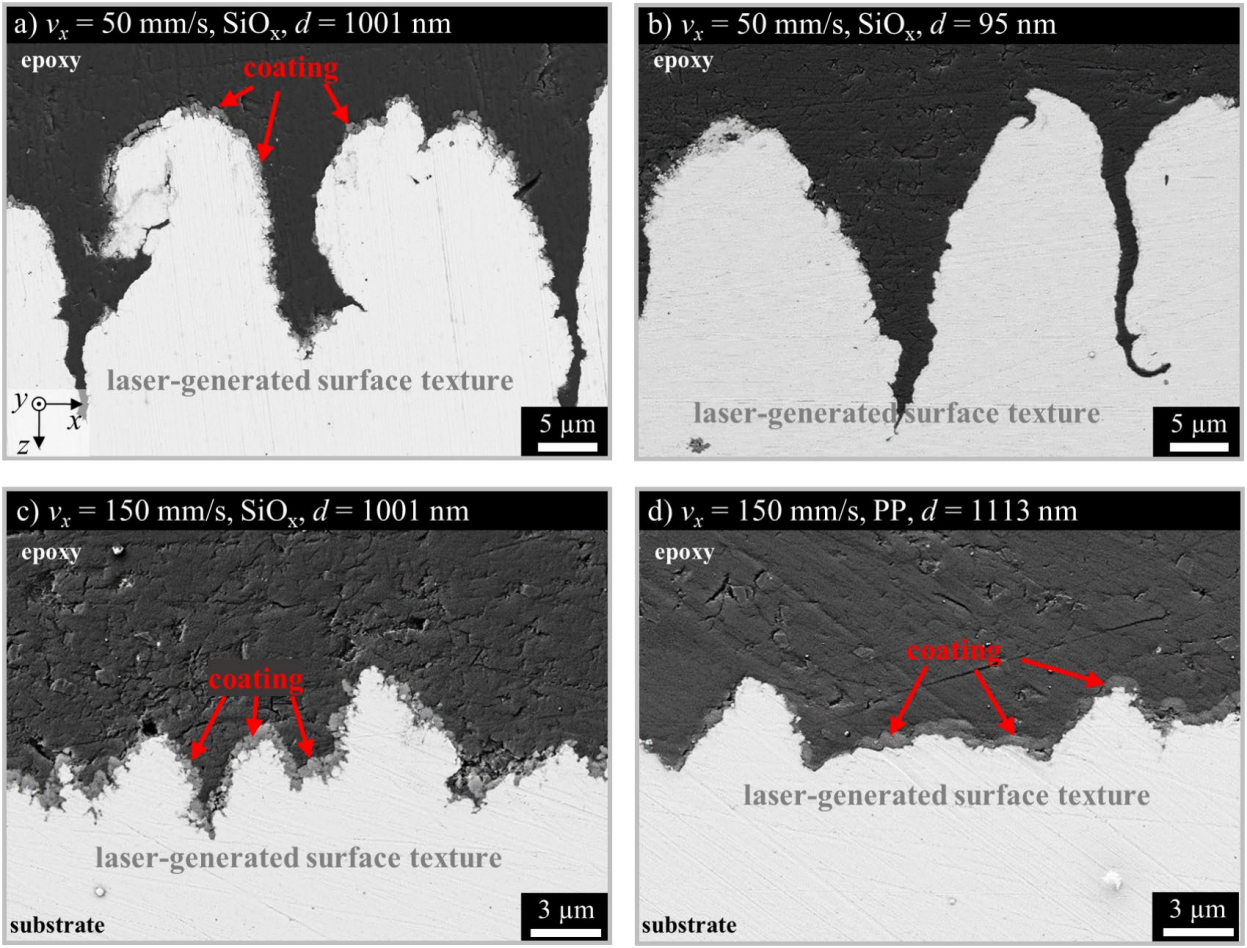
Cross sections of different surface textures on stainless steel obtained by laser texturing with *ϕ*_0_ = 0.65 J/cm^2^ at different scanning speeds *v*_*x*_ and then coated with different coating materials and thicknesses* d*. The coating with a thickness of less than 100 nm could not be clearly differentiated from the epoxy in (**b**).

A coating layer was applied by PECVD to chemically modify the textured surfaces. To evaluate the layer formation after plasma coating of the laser textured samples, the treated samples were embedded in epoxy, cross-sectioned and sanded. Figure 4 shows SEM images of cross sections of different surface textures on stainless steel obtained by laser texturing at different scanning speeds *v*_*x*_ and then coated with different coating materials and coating thicknesses *d*.

The light areas show the laser-generated textures on the surface. The dark areas show the epoxy resin. Between the dark and light areas in Fig. 4a, c, d, the coating is visible on the laser texture as a light grey layer, which is marked with red arrows for better illustration. The applied layers have a thickness of up to 1 µm with local variations of the thickness. However, the majority of the surface is coated, even the side walls of some of the protrusions and the valleys between the protrusions. It is possible that during the preparation of the cross sections, the epoxy may have smeared over the coating due to different sanding directions and the coating may not be clearly visible in all areas. Thin layers with a thickness in the range of 100 nm could not be detected or differentiated from the epoxy with this method, see Fig. 4b. The different coatings SiO_x_ and PP show a similar appearance on comparable textures in terms of thickness and homogeneous distribution of the coating (cf. Fig. 4c, d). These results show that the surface topography can be modified by the LasPlas process and additional layers can be applied to this surface topography.

## Wettability of metal surfaces after advanced surface treatment

### Time dependent wetting behavior and attainable contact angles of surfaces treated by either laser texturing or plasma coating

**Fig. 5:**
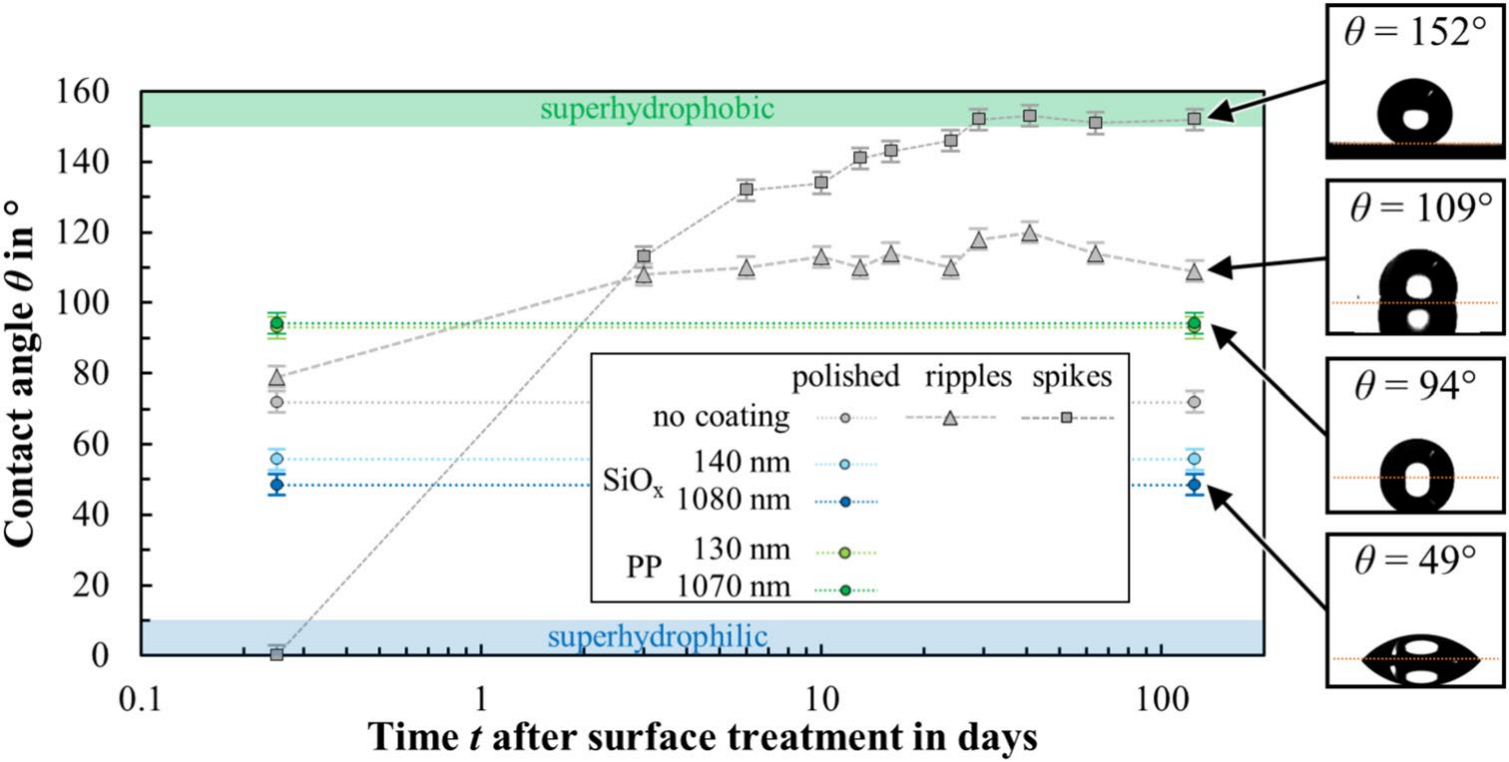
Measured contact angle of DI water as a function of time after surface treatment for different surface topographies (polished, ripples, spikes) with either no coating, a thin coating (130 to 140 nm) or a thick coating (1070 to 1080 nm) of different coating material (SiO_x_, PP). The insets to the right of the plot show exemplary images of the water droplets on the surface and the corresponding contact angle. The contact angle areas of superhydrophobic (*θ* > 150°) and superhydrophilic (*θ* < 10°) states are indicated in green and blue, respectively.

The results of the wetting measurements for steel surfaces treated by the individual surface treatments (laser texturing or plasma coating) are presented in the following and compared with the untreated initial surface, i.e. polished and uncoated. Figure 5 shows static contact angles of DI water on these surfaces as a function of time after surface treatment with either laser texturing (ripples, spikes) or plasma coating with different materials (SiO_x_, PP) and different coating thicknesses.

Polished surfaces without laser texture or coating (Fig. 5, grey circles) showed a hydrophilic state with a contact angle of *θ* = 72° during the observation time of 125 days. With a thin SiO_x_ coating (*d* = 140 nm), the contact angle was be reduced to *θ* = 56° (Fig. 5, light blue circles). Increasing the coating thickness (*d* = 1080 nm) only led to a slight further decrease in the contact angle to *θ* = 49° (Fig. 5, dark blue circles). With a thin PP coating (*d* = 130 nm) on the polished surface, the contact angle was be increased from *θ* = 72° to *θ* = 93° (Fig. 5, light green circles), i.e. the wetting behavior changed from hydrophilic to hydrophobic. Surfaces with a thick PP coating (*d* = 1070 nm) exhibited a contact angle of *θ* = 94° (Fig. 5, dark green circles), which is a slight increase within the measurement uncertainty of ± 3°. By selecting the appropriate coating material, hydrophobic or hydrophilic wetting behavior can be thus obtained permanently on polished samples, but not in the “super-”wetting states.

Surfaces with laser textured ripples (Fig. 5, grey triangles) exhibit a contact angle of *θ* = 79° immediately after texturing (*ϕ*_0_ = 0.65 J/cm^2^, *v*_*x*_ = 4800 mm/s) which is slightly higher than the contact angle on the untreated surface. The contact angle then increases over time, fluctuating in the hydrophobic range of 109° ≤ *θ* ≤ 120° only 3 days after the surface treatment. Surfaces with spikes (Fig. 5, grey squares) are superhydrophilic with a contact angle of *θ* ≈ 0° (not measurable) immediately after laser texturing (*ϕ*_0_ = 0.65 J/cm^2^, *v*_*x*_ = 50 mm/s). The contact angle then increases continuously over 30 days and is then constantly in the superhydrophobic range of 151° ≤ *θ* ≤ 153°. As previously stated in the introduction, the underlying cause of the increasing contact angle can be attributed to the ageing and contamination of the reactive laser-textured surface^4^. Compared to the coatings, the creation of a spike texture makes it possible to achieve “super-”wetting states, but the superhydrophilic state is not stable over time and the superhydrophobic state is only reached after around 30 days.

The results demonstrate that the individual processes of laser texturing and plasma coating face limitations with regard to achieving temporal stability, instant availability, adjustability and “super-”wetting states. In the following, the limitations of the individual processes are overcome by sequential combination of laser texturing and plasma coating.

### Instant and permanent superhydrophilic steel surfaces with high wettability by combined laser texturing and plasma coating

**Fig. 6:**
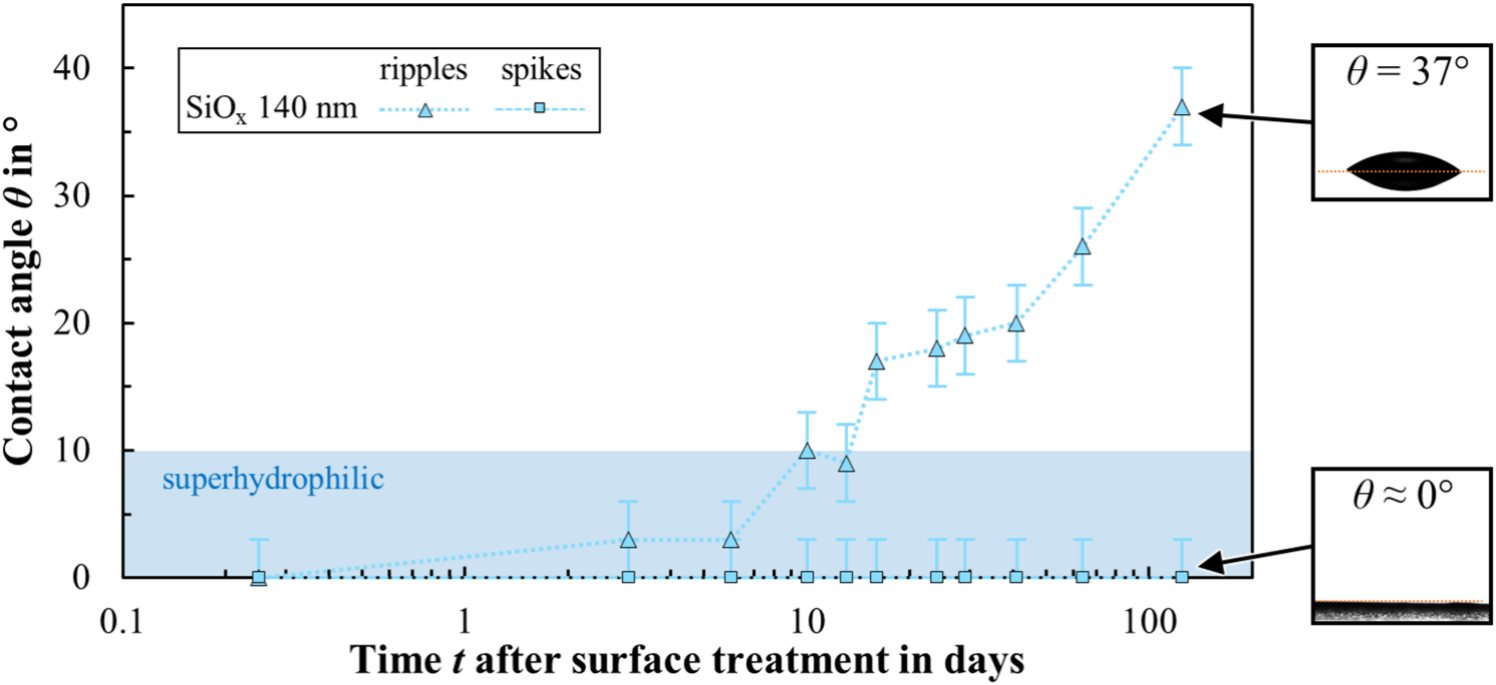
Measured contact angle of DI water as a function of time after surface treatment for different surfaces (ripples, spikes) obtained from laser texturing and plasma coating of a thin SiO_x_ coating with a thickness of *d* = 140 nm. The insets to the right of the plot show exemplary images of the water droplets on the surface and the corresponding contact angle. The contact angle area of a superhydrophilic state (*θ* < 10°) is indicated in blue.

The fabrication of metal surfaces with high wettability, i.e. superhydrophilic surfaces, was investigated using different laser textures (ripples, spikes) and the SiO_x_ coating with different thicknesses (140 nm, 1080 nm). Figure 6 shows the measured static contact angles of DI water on these surfaces as a function of time after surface treatment. Some examples of camera images of the applied droplets are shown on the right, which were used to measure the static contact angle. For reasons of overview, the results for textured surfaces for the coating thickness of *d* = 1080 nm are not shown in Fig. 6; nevertheless, the mentioned surfaces show a similar behavior to the samples coated with a thickness of *d* = 140 nm.

A thin coating of SiO_x_ instantly gives surfaces with either ripples (Fig. 6, light blue triangles) or spikes (Fig. 6, light blue squares) superhydrophilic properties with a contact angle of *θ *≈ 0°. The SiO_x_ layer on the laser textures increases the surface energy and thus enhances wettability of the treated surface. However, the contact angle of the coated ripples increases again over time and is still in the hydrophilic range of 37 after 125 days. Even if the reactive laser-textured surface has been covered by the hydrophilic coating, the contact angle can still increase due to the deposition of contaminants from the ambient air. The contact angle of the coated spikes remained at ≈ 0° for the duration of the test shown in Fig. 6. However, an increased contact angle of *θ* = 78° was determined in a measurement more than one year after the surface treatment. Here, too, the deposition of contaminants on the surface was assumed, as after cleaning the sample for 3 min in acetone in an ultrasonic bath, a contact angle of ≈ 0° was again observed and remained at this value even 14 days after the cleaning process.

The aforementioned coated samples were all coated approximately one hour after laser texturing. From a production point of view in an industrial environment, it is also interesting to know within how long the coating must be applied after laser texturing in order to achieve the corresponding superhydrophilic properties. Therefore, a surface textured with spikes (initially without coating) was exposed to air for 30 days until the surface was superhydrophobic with a contact angle exceeding 150° (cf. Fig. 5, grey squares). The sample was then coated with SiO_x_ with a thickness of 140 nm. After coating, the surface was superhydrophilic again with a contact angle of *θ *≈ 0°, i.e. the coating “covered” the surface that had been chemically modified by aging at ambient atmosphere. The time elapsed between laser texturing and coating therefore appears to be irrelevant for the superhydrophilic wetting effect.

**Fig. 7:**
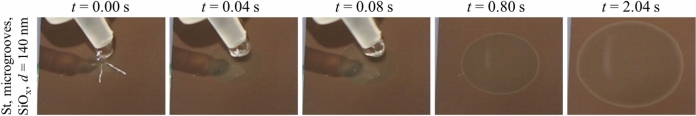
Time-dependant spreading of a drop of DI water at different times after application on a laser-textured stainless steel sample (microgrooves, *ϕ*_0_ = 0.65 J/cm^2^, *v*_*x*_ = 150 mm/s) coated with SiO_x_ (*d* = 140 nm).

Figure 7 shows the distribution of a water droplet (10 µℓ) applied with a pipette on a steel surface treated with the LasPlas process at different times *t* after application.

The laser-textured microgrooves were coated with a thin layer of SiO_x_ (*d* = 140 nm). The image at *t* = 0.00 s shows the recorded frame at the moment of droplet application. At *t* = 0.00 s, the droplet initially contacts the surface, and within 0.04 s, it has already begun to spread significantly. This rapid expansion indicates a highly hydrophilic nature of the surface, where the high surface energy drives immediate wetting. Over time, the droplet continues to expand, forming a thin and nearly circular liquid film. By *t* = 2.04 s, the droplet has fully spread, covering a large surface area with a minimal contact angle* θ* ≈ 0°. This final state confirms complete wetting, which is an important characteristic of superhydrophilic surfaces.

The LasPlas surface treatment, consisting of a rough laser texture and a thin glass coating, therefore provides an immediate and permanent superhydrophilic and highly wettable surface on steel.

### Instant and permanent superhydrophobic steel surfaces with high liquid repellence by combined laser texturing and plasma coating

**Fig. 8:**
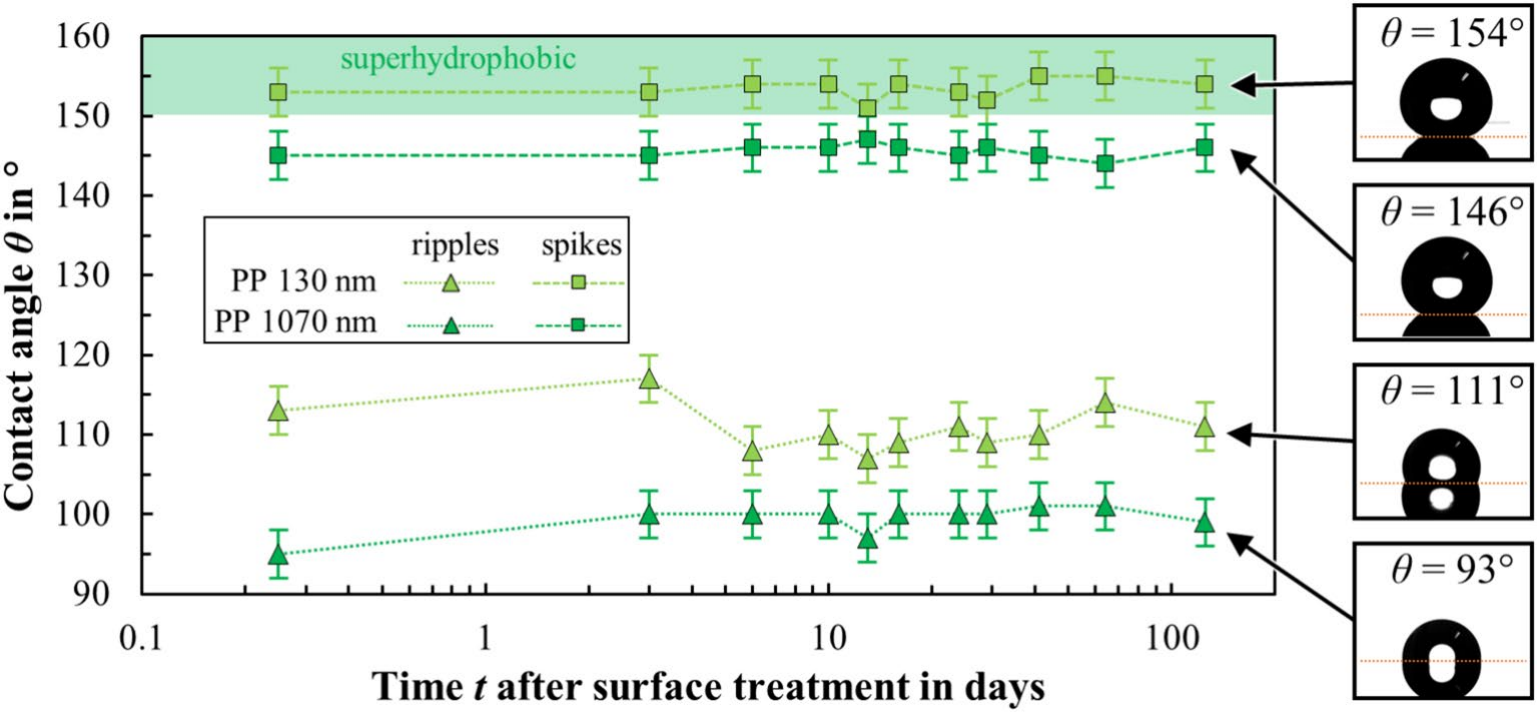
Measured contact angle of DI water as a function of time after surface treatment for different surfaces (ripples, spikes) obtained from laser texturing and plasma coating of a thin (*d* = 130 nm) or thick (*d* = 1070 nm) layer of PP. The insets to the right of the plot show exemplary images of the water droplets on the surface and the corresponding contact angle. The contact angle area of a superhydrophobic state (*θ* > 150°) is indicated in green.

The fabrication of metal surfaces with high repellence, i.e. superhydrophobic surfaces, was investigated using different laser textures and the PP coating with different thicknesses. Figure 8 shows the measured contact angles of DI water on these surfaces as a function of time after surface treatment. Some examples of camera images of the applied droplets are shown on the right, which were used to measure the static contact angle.

A thin coating of PP (*d* = 130 nm) gives the surface with ripples immediately hydrophobic properties with a contact angle in the range of 109° ≤ *θ* ≤ 117° (Fig. 8, light green triangles). The PP layer on the laser textures reduces the surface energy and thus reduces wettability of the treated surface. A thicker coating of PP (*d* = 1070 nm) also immediately gives the surface with ripples hydrophobic properties, however, with a lower contact angle in the range of 95° ≤ *θ* ≤ 101° (Fig. 8, dark green triangles). The reason for this decrease is probably the ratio of the layer thickness to the dimensions of topography of the laser texture. The application of the approximately 1 µm thick coating on the fine ripple-shaped texture with an average roughness *S*_a_ = 0.29 µm leads to a smoothing of the surface topography, which is why this surface then behaves similarly to the polished and coated surface (Fig. 5, dark green circles).

A thin coating of PP immediately gives the surface with spikes permanent superhydrophobic properties with a contact angle in the range of 151° ≤ *θ* ≤ 155° (Fig. 8, light green squares). From an application point of view in particular, the immediate production of long-lasting wetting conditions without additional storage for a few days is relevant for the immediate and reliable functionality of a surface. A thicker coating of PP yields immediately hydrophobic properties with a significantly lower contact angle in the range of 144° ≤ *θ* ≤ 147° (Fig. 8, dark green squares). As with the ripples, the influence of the thick coating on the surface topography is assumed to be the cause of the reduced contact angle. Since superhydrophobicity relies on a combination of micro- and nanoscale roughness to trap air pockets, the thick coating presumably smoothed the nanoscale roughness. Additional SEM images may provide further insight in this regard.

**Fig. 9:**
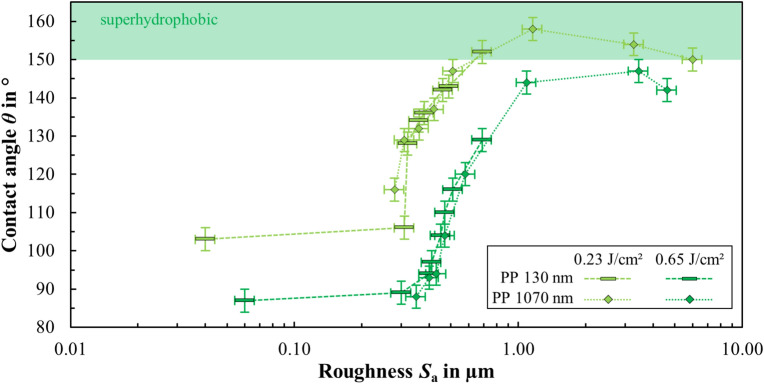
Measured contact angle of DI water as a function of surface roughness for stainless steel surfaces textured with different peak fluences (and scanning speeds) and coated with different thicknesses of PP. The contact angle area of a superhydrophobic state (*θ* > 150°) is indicated in green.

A possible correlation between contact angle *θ* and roughness *S*_a_ can be seen within a series of measurements (thin or thick coating). Figure 9 shows the contact angle measured 371 days after surface treatment as a function of surface roughness for different combinations of surface topographies (produced by different peak fluences and scanning speeds) and different coating thicknesses of PP on stainless steel.

Figure 9 shows, that the laser texturing of HSFL (data point at *S*_a_ = 0.04 µm) on steel surfaces does not lead to a large increase in the average roughness (compared to *S*_a_ = 0.03 µm of the polished surface), but it does lead to a significant increase in the contact angle from 72° to 107°. In the range 0.3 µm < *S*_a_ < 1 µm the contact angle increases steeply regardless of whether the laser textured samples are thinly coated with PP (*d* = 130 nm) or thickly coated with PP (*d* = 1070 nm). In the range 1 µm < *S*_a_ < 3 µm the contact angle reaches its maximum and then decreases again for higher roughness values *S*_a_ > 3 µm. There appears to be an optimum roughness at which a maximum contact angle is obtained. The reason for this behavior can be explained using the Wenzel and Cassie-Baxter model. At low roughness, the surface follows the Wenzel state, where water penetrates the surface textures. As roughness increases, the Cassie-Baxter state emerges, where air pockets become trapped beneath the droplet, reducing liquid–solid contact and increasing the contact angle. Beyond a critical roughness, excessive texture size may collapse the Cassie state back to the Wenzel state, reducing the contact angle again.

Superhydrophobic surfaces with contact angles of over 150° were achieved here in the range of roughness of 0.7 µm < *S*_a_ < 3.3 µm. This is relevant since the production of rougher laser textures requires more energy (e.g. higher number of pulses per spot and therefore lower scanning speed *v*_*x*_)^38^ and therefore lower texturing rates are obtained (cf. Fig. 3). Regarding applications, the surfaces should therefore only be roughened just enough to achieve the desired functionality (e.g. a contact angle *θ* > 150°) in order to be able to generate the surfaces with the highest possible texturing rate and thus high throughput. The roughness parameter *S*_a_ can be used as a measurable indicator of whether the desired laser texture, and thus the functionality of the texturing process, has been achieved. Roughness measurement with an LSM is easier to integrate into a laser-based production process than with an SEM, as no vacuum is required. In the case of stainless steel, the highest contact angle of *θ* = 158° was achieved with the laser texture microgrooves (cf. Fig. 2f) coated with either a thin layer of PP (*d* = 130 nm) or PTFE (*d* = 160 nm). When stored in ambient air, this sample still showed superhydrophobic wetting behavior two years after production.

**Fig. 10:**
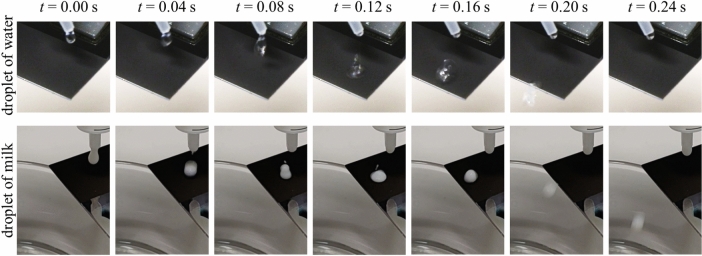
Camera images showing the jumping-off of a droplet of (**a**) DI water and (**b**) full milk at different times after droplet application on a laser-textured and PTFE-coated stainless steel surface. These results demonstrate that only the combined LasPlas process enables dynamic repellence and residue-free removal of complex fluids such as milk and beer - a result not achieved by texturing or coating alone.

The samples with contact angles *θ* > 150° were also exposed to dynamic wetting situations by means of camera-based roll-off measurements. The samples were tilted to an angle of about 10° and droplets (10 µℓ) of different liquids were applied to the surface using a pipette. Based on potential applications in the food industry (provided suitable, food-safe coatings are selected), whole milk (*K Classic*, 3.5% fat, homogenized) and beer (*Wulle*, 5.0% alcohol) were examined in addition to DI water, i.e. liquids that contain fats and lactose or alcohol and carbon dioxide in addition to water. Figure 10 shows the roll-off behaviour of water (top row) and milk (bottom row) on functionalized surfaces at different times *t* after droplet application (columns). For functionalization, the stainless-steel surfaces were textured with microgrooves (*ϕ*_0_ = 0.65 J/cm^2^, *v*_*x*_ = 150 mm/s) and then coated with PTFE (*d* = 160 nm).

The droplets fall from the pipette onto the surface (*t* = 0.08 s), causing the droplets to deform temporarily (*t* = 0.12 s) and then roll or bounce (*t* = 0.20 s) from the surface into the glass beaker below. Even when several droplets were applied, they left no residue on the surfaces. Although only the roll-off tests for DI water and milk are shown in Fig. 10, this also worked for beer. In the case of milk in particular, comparative tests with untreated (polished), only laser-textured (microgrooves) or only coated (PTFE) samples showed that no repellent behavior could be obtained, but that the droplets spread on the surface. Only the combination of laser texturing and plasma coating achieved residue-free roll-off of water, milk, and beer droplets. While many previous studies demonstrate self-cleaning with pure water, our use of complex, real-world fluids such as milk and beer highlights a more application-relevant challenge, revealing that effective liquid repellence in practical settings requires both tailored topography and optimized surface chemistry, which is a combination often overlooked in generic self-cleaning demonstrations.

The LasPlas surface treatment, consisting of a rough laser texture and a thin polymer coating, therefore enables immediate and permanent superhydrophobic and liquid-repellent surfaces on steel.

### Application of laser texturing and plasma coating on copper and a titanium alloy

**Fig. 11:**
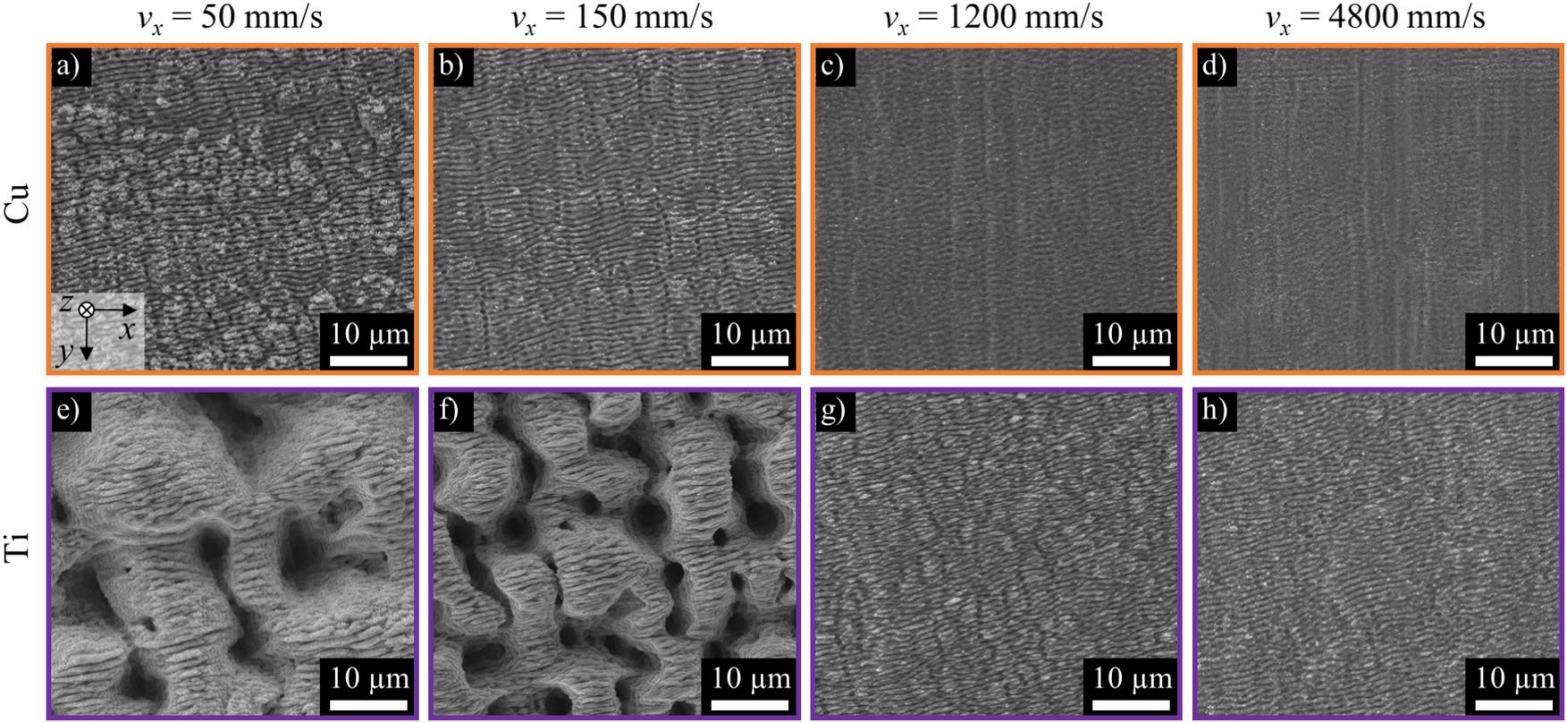
SEM images of surface textures after laser surface texturing with different scanning speeds on stainless steel on copper (Cu, a to d) and the titanium alloy Ti64 (Ti, e to h). *λ* = 1030 nm, *τ* = 260 fs, *f*_P_ = 200 kHz, *d*_0_ = 120 ± 5 µm, *ϕ*_0_ = 0.65 J/cm^2^, *p*_*y*_ = 24 µm,* n* = 1.

In the following, the great potential of the LasPlas process in terms of modifying the wetting of stainless steel is also demonstrated for the other metals copper and titanium. Figure 11 depicts SEM images of laser-textured surfaces copper (Cu, upper row) and Ti64 (Ti, lower row) textured with a peak fluence of *ϕ*_0_ = 0.65 J/cm^2^ and varying scanning speeds *v*_*x*_ in the range of 50 mm to 4800 mm/s.

LIPSS developed on the copper surface over the investigated scanning speeds. Although textures with increased roughness also occur on copper at lower scanning speeds, no rough spikes or holes were observed even at the lowest scanning speed of *v*_*x*_ = 50 mm/s (Fig. 11a). This is due to the low absorption and high thermal conductivity of copper, which results in less energy being coupled in on the one hand and the residual energy dissipating from the surface into the surface in a short time on the other. The formed textures of the titanium alloy are similar to those of stainless steel (cf. Fig. 2) at the same peak fluence and scanning speed due to similar optical and thermophysical properties. The rougher structure of the titanium alloy at *v*_*x*_ = 150 mm/s (Fig. 11f) compared to the texture of stainless steel (Fig. 2f) was probably due to the higher initial roughness of the titanium sheet, which led to higher energy coupling and thus higher temperature.

**Fig. 12:**
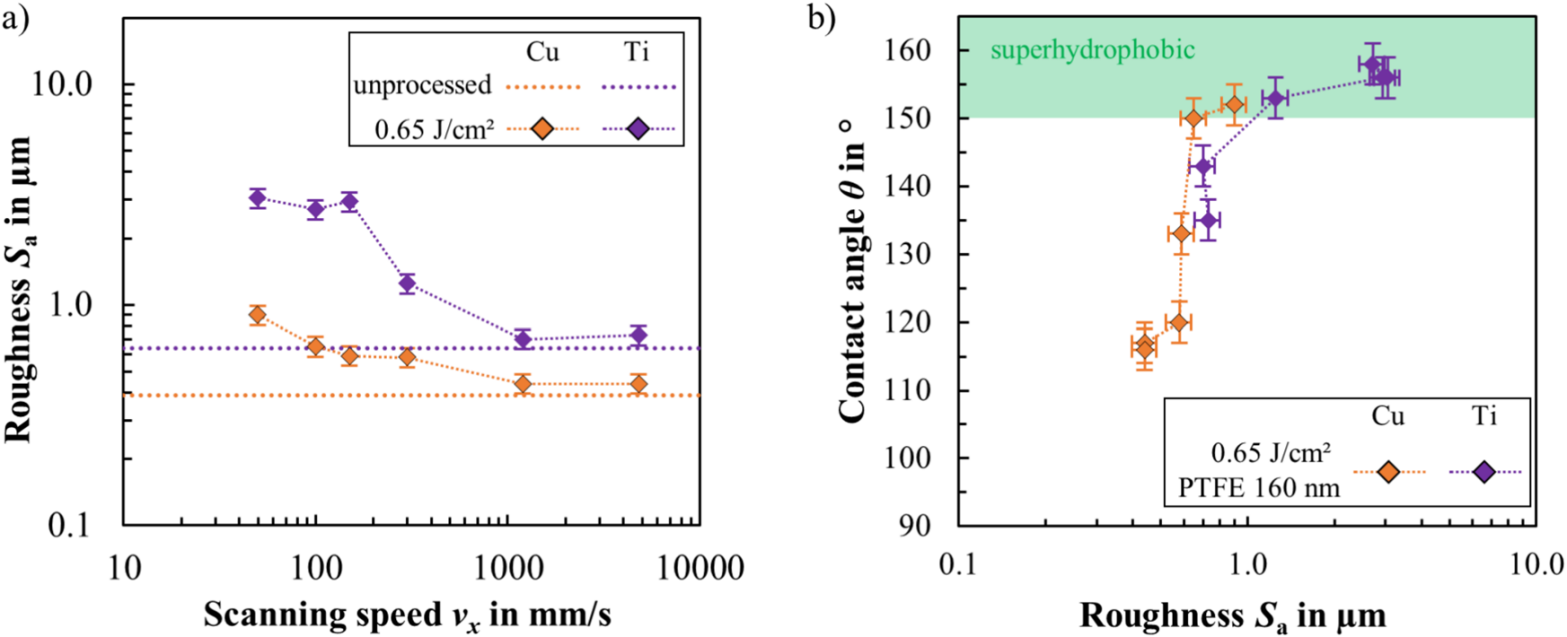
(**a**) Measured surface roughness *S*_a_ of the laser textured copper (Cu) and titanium (Ti) surfaces as a function of scanning speed *v*_*x*_. The roughness of the unprocessed initial surfaces is shown as dotted lines. (**b**) Measured contact angle of DI water as a function of surface roughness of copper and titanium surfaces textured with different scanning speeds and coated with PTFE. The contact angle area of a superhydrophobic state (*θ* > 150°) is indicated in green.

Figure 12a shows the roughness values *S*_a_ of textured copper and titanium surfaces as a function of the scanning speed *v*_*x*_. Orange data points represent copper and blue data points represent the titanium alloy. The roughness values of the unprocessed initial surfaces are shown as dotted lines in the corresponding colors. Figure 12b shows the correspondent contact angles *θ* as a function of the roughness *S*_a_ of textured copper and titanium surfaces that were subsequently coated with a thin layer of PTFE (*d* = 160 nm).

As with stainless steel, low scanning speeds lead to the surfaces with the highest roughness values. With increasing scanning speed, the roughness decreases and approaches the initial roughness of the unprocessed sheets (Fig. 12a). It can be seen that the initial roughness of the surface to be textured has a major influence on the resulting roughness after texturing. The less rugged surface topography of copper shown in Fig. 11 is also evident in the lower roughness values obtained (Fig. 12, orange diamonds) compared to those of the titanium alloy (Fig. 12, violet diamonds).

Figure 12b shows that the contact angle *θ* increases with increasing roughness *S*_a_. A particularly steep increase can be seen for copper in the range 0.5 µm ≤ *S*_a_ ≤ 0.7 µm. Superhydrophobic surfaces with contact angles of over 150° were achieved here for copper and titanium in the range of roughness of 0.7 µm < *S*_a_ < 3.1 µm. These results correspond to those for stainless steel, indicating that maximum contact angles occur with laser textured and coated metals in this optimum roughness range.

**Fig. 13:**
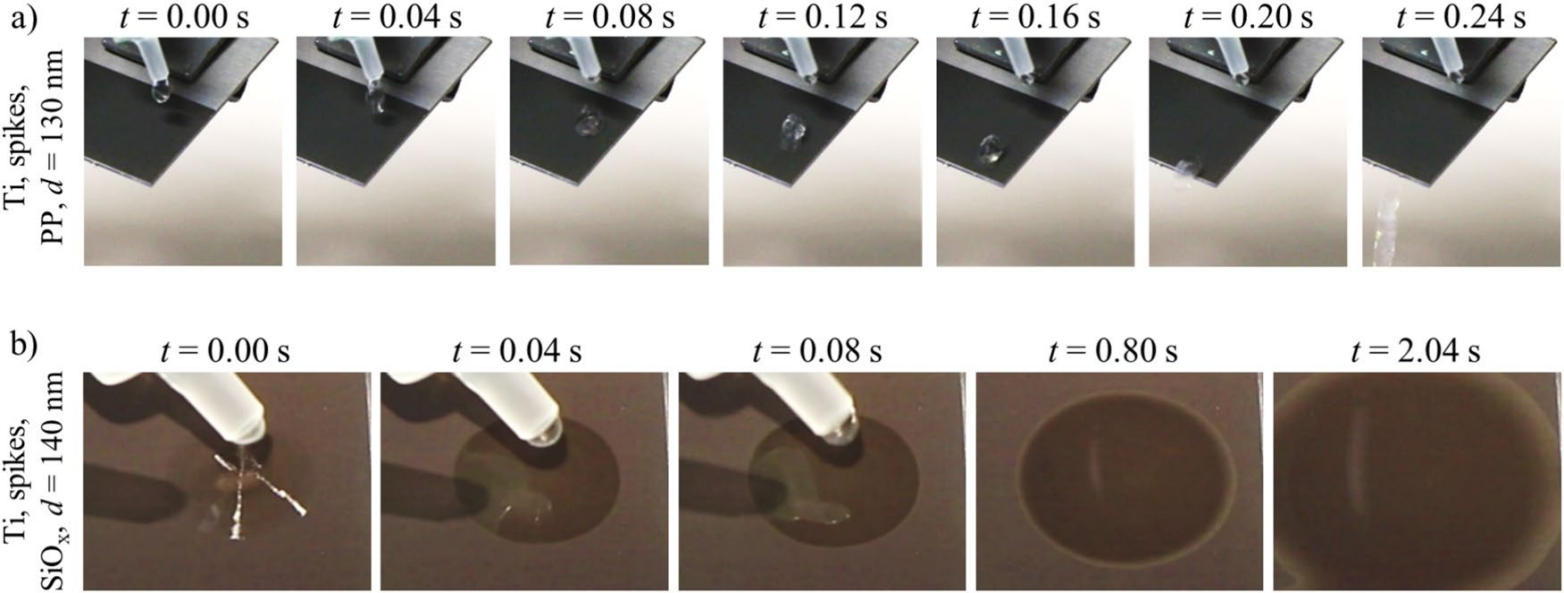
Camera images of LasPlas-treated titanium surfaces showing (**a**) the jumping-off of a droplet of DI water obtained by spike textures and the thin polymer coating and (**b**) the spreading obtained by spike textures and the thin glass coating.

Extreme wetting conditions were also achieved on copper and titanium with rough surface textures and thin coating layers. Figure 13a, b shows the different wetting behavior of LasPlas-treated titanium surfaces with DI water (10 µℓ) with the same rough spikes texture but with different coating materials.

Figure 13a shows that the thin coating layer of PP (*d* = 130 nm) on the titanium surface covered with spike textures caused the droplets to roll or bounce from the surface without leaving residues on the surface. On the contrary, the thin coating layer of SiO_x_ (*d* = 140 nm) resulted in a strong wetting of the surface, with the droplet spreading immediately on the treated surface (Fig. 13b). This approach also worked for roughened copper surfaces and corresponding coatings (not shown here).

The LasPlas process therefore enables superhydrophobic or superhydrophilic surfaces to be created instantly and with longevity under ambient storage on various technically relevant metals such as stainless steel, copper and titanium through a targeted adjustment of the rough laser texture and thin coating material.

## Conclusion

The combination of laser texturing and plasma coating allows tunable, permanent and “super-”wettability states across multiple metals and fluids after the surface treatment. The combination of laser surface texturing and PECVD presents a powerful and flexible approach to achieving and stabilizing extreme wetting properties. Laser texturing introduces the necessary roughness to amplify wetting effects, while plasma coatings tune the chemical interactions to achieve desired contact angles. Together, these methods enable the fabrication of superhydrophilic or superhydrophobic surfaces with functional longevity.

Instant and permanent superhydrophilic metallic surfaces with high wettability (contact angle *θ* < 10°) were achieved by rough textures (e.g. microgrooves, spikes) covered by a thin glass coating (SiO_x_, ≈ 100 nm thickness). During long-term storage (> 1 year), contamination from the ambient air led to an increase in the contact angle. However, this increase could be reversed by cleaning with acetone in an ultrasonic bath, making the sample superhydrophilic again in the long term. In addition, superhydrophobic samples without a coating could be made superhydrophilic again by coating with the thin glass layer.

Instant and permanent superhydrophobic metallic surfaces with high liquid repellence (contact angle *θ* > 150°) were achieved by rough textures (microgrooves, spikes) and covered by a thin polymer coating (PP or PTFE, ≈ 100 nm thickness). Due to the optimum roughness in the range of 1 to 3 µm, the surfaces should only be roughened just enough to achieve the desired functionality (e.g. a contact angle *θ* > 150°) in order to generate the surfaces with the highest possible texturing rate and thus high throughput. In addition to DI water, the surfaces functionalized in this way also exhibited highly repellent properties without residues for liquids such as milk and beer, which makes this approach of laser texturing and plasma coating also particularly useful for applications using solutions or emulsions.

Future work will need to investigate the thermal and chemical stability as well as the resistance of the LasPlas surfaces to UV radiation^60^ or mechanical abrasion^61^ by systematic aging studies under harsher or application-specific environments. Furthermore, large-area functionalization^40,41^ and integration into production environments are required to demonstrate the scalability for industrial applications.

## Data Availability

The data that support the finding of this study are available from the corresponding author upon reasonable request.
